# Comparative Analysis of Facial Coloration between Introduced and Source Populations of the Red Wood Ant *Formica paralugubris*

**DOI:** 10.3390/insects13121137

**Published:** 2022-12-09

**Authors:** Filippo Frizzi, Laura Buonafede, Alberto Masoni, Paride Balzani, Giacomo Santini

**Affiliations:** 1 Department of Biology, University of Florence, Via Madonna del Piano, 6, Sesto F.No., 50019 Florence, Italy; 2 Faculty of Fisheries and Protection of Waters, South Bohemian Research Center of Aquaculture and Biodiversity of Hydrocenoses, University of South Bohemia in České Budějovice, Zátiší 728/II, 38925 Vodňany, Czech Republic

**Keywords:** phenotypic trait, red wood ants, alien species, relative warp analysis, body color

## Abstract

**Simple Summary:**

Animals’ body coloration can be implied in several ecological and behavioral processes, but it is understudied in ants. In this study, we compared the differences between the facial coloration of sixty-year-old imported populations of the red wood ant *Formica paralugubris* and their source populations, using a shape-based analytical approach for the first time. We found that, except for a case we discuss, the facial coloration is overall similar between introduced and source populations, suggesting a stable genetic background. Interestingly, we found that the large difference in the habitat in which populations naturally dwell or were established—forests dominated by spruce, beech, fir, and a mix of these—has a low effect on shaping this trait, as we might expect from previous studies. However, the within-population variability suggests that facial coloration is probably affected by very localized external factors at the population and nest level. Finally, we found that the ant size affects the trait, a result in line with previous studies.

**Abstract:**

The variation in the typical black-reddish color of red wood ants (*Formica rufa* group) has been recently suggested as a good indicator of habitat quality, being dependent on environmental conditions. However, the relative contribution of external factors and heritability in shaping this trait is poorly investigated. In this study, we compared the facial coloration of workers from four introduced populations of *Formica paralugubris* with those of the two Alpine populations from which they had been taken. We used a Relative Warp Analysis to describe the variations in the shape of this trait. We expected each introduced population to be more similar to its population of origin if the color pattern was predominantly genetically determined. On the contrary, due to the considerable differences in habitat type and climate between the Alps and the Apennines, we expected to observe differences between the introduced population and their origin population if the coloration was mostly environmentally determined. With one exception that we discuss, the results showed that ants from the two source populations had different phenotypes, and that the introduced populations had a shape similar to the population of origin, suggesting a stable genetic background. Surprisingly, the habitat type seems to have a less clear effect, even if within-population differences suggest the influence of very localized environmental factors. Finally, we found that the facial coloration shape is affected by the ant’s size, a result in line with previous studies.

## 1. Introduction

Body color is an essential phenotypic character that may have a central adaptive role in the relationships of organisms with the environment (Stuart-Fox and Moussalli, 2008). In insects, body color is involved in several behavioral and ecological vital aspects, such as intra- and interspecific signaling, mimicry, thermoregulation, and even protection against pathogens [1,2,3,4,5,6]. Hymenopterans, given their environmental and economic importance, have always been in the spotlight of ecological and behavioral research, and interest in the role of body coloration is increasing [5]. Many studies focused on wasps, whose facial and body colors can have a role in visual social communication or in signaling the status of the opponent during aggressive interactions among conspecifics [7,8,9,10]. In stingless bees (*Melipona* spp.), bumblebees (*Bombus* spp.), and paper wasps (*Polistes* spp.), coloration is also involved in thermoregulation, with dark colors of the body that vary with latitude and body size in order to cope with changing temperatures [3,11,12].

Quite surprisingly, there have been few studies on the importance of body coloration in ants, although these insects are widespread over most terrestrial habitats and are key organisms in many ecological dynamics [13]. We know, for example, that in the genus *Diacamma,* the body color is one of the most relevant traits defining sexual dimorphism, [14] and that in the Saharan silver ant *Cataglyphis bombycina* (Roger, 1859), the highly reflective silvery color of the hair can mirror a large part of the solar radiation, thus helping the ants in thermoregulation [15]. A recent large-scale study on the *Pheidole* genus shows that the larger castes of the species inhabiting regions with low temperatures and high precipitation are darker, probably because of several co-acting factors [16]. At the community level, it has been found that in a rainforest, the coloration of the species is stratified, with darker species being more common in the canopy and lighter species closer to the soil [17].

Recently, several studies have focused on the coloration of species belonging to the *Formica rufa* group, cold-adapted ants also known as red wood ants—or simply RWAs—because of their typical black-reddish coloration of the body [18]. These ants build typical large nest mounds using understory materials, such as fir needles, resin granules, small pebbles, and soil particles, which may affect the soil chemistry and host many symbiotic invertebrates [19,20,21,22,23]. The European RWA species are widespread in central to northern Europe and in the mountains of southern countries [18,24]. Because of their top-ranking position in the ecological dominance hierarchies, they have a relevant influence on the ecological dynamics of the habitats in which they dwell, and a concern about their complex conservation status has been recently pointed out in Europe [25,26]. The body coloration of RWAs, though overall similar, may show interspecific differences and can be a useful trait for their identification [18]. For example, the meadow ant *Formica pratensis* (Retzius, 1783), the species inhabiting the southernmost regions among all the European species, has on average a darker color than the other species [27], so much so that some populations have been classified for decades as belonging to a different subspecies (*Formica pratensis nigricans*, Emery, 1909) or even to a separate species (*Formica nigricans*, Bondroit, 1912) [28] (in Italian), a classification that has currently been abandoned [24,29].

Body coloration may be variable even among conspecifics. In *Formica rufa* (L.), the color patterns are related to the size of workers, with small individuals having a relatively darker head and thorax than large ones [30]. Interestingly, facial coloration patterns show a discrete variability, contrarily to a more continuous variation in the thorax color [30]. In *Formica aquilonia* (Yarrow, 1955), exposure to pathogens may trigger a melanization reaction, consisting in an increase in the body’s concentration of melanin, which has anti-pathogenic properties, thus making the workers darker [31]. Because of this dependency on environmental factors, the use of color variation in RWAs as an environmental indicator has been recently encouraged [32]. However, to date, the way the variability of body coloration is related to genetic and external factors in these ants is a poorly explored matter [5]. A recent study on *F. rufa* did not find any correlation between the variability of coloration and the kinship of workers [33], but a phylogenetic study on the heritability of this trait is lacking so far.

In this study, we performed a comparative analysis of the facial coloration among different populations of the RWA *Formica paralugubris* (Seifert, 1996). This dominant, highly polydomous, and polygynous forest-dwelling species has been introduced into many Apennine sites—where it was absent—from different alpine populations during the early second half of the 20th century, in order to use it as a biological control agent for several forest insect pests [34,35] (in Italian). After an initial decline, the introduced populations started to expand, raising concerns about their impact on local communities [36,37,38]. Two features helping the adaptability of this species to the different Apennine environmental conditions are the plasticity of its diet and the ability to use different nest mound materials according to the habitat in which they dwell [39,40]. Here, we used a landmark-based morphological approach to investigate the interplay between inherited and acquired components of facial coloration. To this end, we compared this trait between four introduced populations in the Apennines and their two source populations in the Alps. In particular, we aimed to answer two research questions: (1) Is this trait heritable and conservative? (2) Do different habitat conditions determine changes in the coloration pattern of the imported populations? If color is fully heritable, we can hypothesize that the color pattern of the imported populations would reflect that of their respective source population. However, if habitat features strongly affect body coloration up to override the inherited component, we may expect the Apennine populations to differ from the population of origin in the Alps and to show similarities correlated with the type of habitat they occupy. Building on previous studies, we expected that the main factor affecting the color variability was the habitat type, with the common origin being less determinant.

## 2. Materials and Methods

### 2.1. Sites and Sampling Design

**Figure 1:**
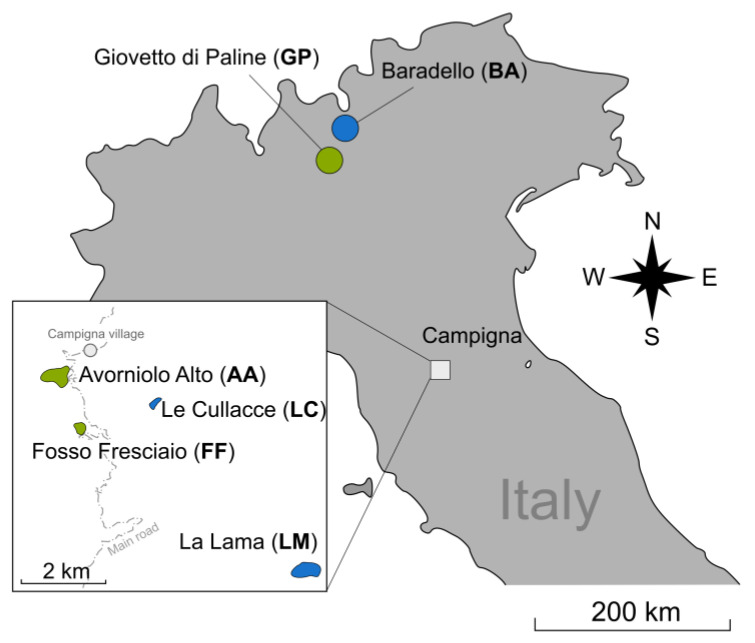
Map of the two original Alpine and the four imported populations of *Formica paralugubris*. The same color denotes the same origin.

All sampling sites were located in Italy (Figure 1). The two alpine-source populations are those of the Giovetto di Paline Nature Reserve (GP, Bergamo, and Brescia provinces, 45°58′00″ N, 10°08′30″ E) and Baradello mountain (BA, 46°08′30″ N, 10°09′40″ E), straddling the municipalities of Corteno Golgi and Aprica (Brescia province). The four Apennine-imported populations are all included in the Campigna Biogenetic Nature Reserve (43°52′20″ N, 11°44′50″ E), embedded in the Foreste Casentinesi, Monte Falterona e Campigna National Park. The populations of Avorniolo Alto (AA) and Fosso Fresciaio (FF) were introduced from the population in GP, whereas the populations of La Lama (LL) and Le Cullacce (LC) originated from the population in BA. The AA, FF, and LL populations were introduced in 1958, whereas the LC population was introduced in 1961 [35,36].

In both the Alpine sites, the forest is dominated by spruce (*Picea abies* (L.) H. Karst., 1881), with the co-occurrence of firs (*Abies alba*, Mill., 1759) in GP and larches (*Larix decidua* Mill., 1768) in BA. As for the Apennine habitats, in AA, the forest is dominated by firs with sparse beeches (*Fagus sylvatica*, L. 1753), whereas in FF, it is the reverse. In LC and LL, the forest is mixed, dominated by both firs and beeches. For each of the six populations, we collected ten worker ants from ten nests, for a total of 600 ants sampled. We selected medium-sized nests of similar size (~1000 l), separated by at least 25 m and not connected by ant trails. To verify that all ants were *F. paralugubris*, two ants per nest were genetically checked [41]. Ants were transferred to the laboratory and stored at −20 °C. A scalpel was used to excise the head and the antennae. The heads were then fixed on a paperboard with double-sided adhesive tape and placed under a binocular microscope. The magnified image was photographed using a Nikon Coolpix^®^ (13 Mpixel resolution) camera with a specific adaptor. We took care that heads were not inclined to prevent distortion in the frontal view. A metric reference was added for each sample as a scale.

### 2.2. Morphologic Data Collection and Statistical Analyses

**Figure 2:**
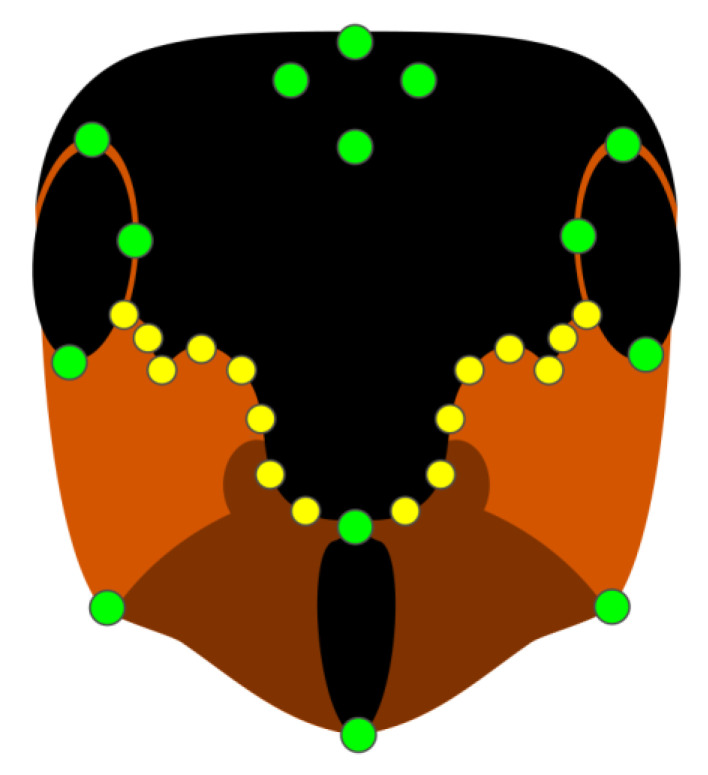
Schematic representation of landmarks (green points) and semi-landmarks (yellow points) used in the analysis.

To assess the shape of the facial coloration, we used the Relative Warp Analysis procedure [42]. In each picture, we placed 14 landmarks in correspondence to fixed points of the head (eyes, frontal and posterior ends of the clypeus, ocelli, and joints between the head and mandibles, Figure 2). Then, we placed 16 sliding semi-landmarks, which followed the shape of the red-black border between the eyes and clypeus, eight per side (Figure 2). Semi-landmarks are allowed to slide along the curve drawn by the color border, a method permitting a reduction in the error due to uninformative shape variations [43]. Landmarks and semi-landmarks of each sample were superimposed in a common coordinate system, and the variability due to the differences in size and orientation was limited by applying a Generalized Procrustes Analysis (GPA) [44]. Landmark-described shapes were then fitted by a thin-plate spline (TPS) interpolation function, which resulted in a bending energy matrix summarizing deformations against a reference configuration [45]. On the eigenvalues resulting from an eigenanalysis of this matrix (i.e., partial warps), a Principal Component Analysis (PCA) was applied, whose components were the relative warps [42], which were subsequently used in multivariate analyses. The whole procedure was performed using tpsRelW, tpsSplin, and tpsUtils software [46].

Finally, since the color pattern can be influenced by the worker size, for each sample, we measured the maximum width of the head above the eyes as a proxy of the body size following Skaldina and Sorvari [30], using the ImageJ software [47]. All the measurements were taken by the same observer, who was blind with respect to the origin of the samples.

Relative warps were used as variables in a multivariate Partial Least Square Discriminant Analysis (PLSDA), which treats the dataset as in a PLS regression—by decomposing the data matrix according to the two first principal components—and then performs a discriminant analysis. This method manages the collinearity of the variables [48] and allows for the exclusion of the irrelevant variables from the dataset by calculating the Variable Importance in Projection (VIP) score. This score depends on the explanatory power of predictors in the PLSDA projection, and if greater than one, the variable can be considered relevant [49]. We selected variables having a VIP score >1 in at least one of the two principal components, and we used these to calculate a Euclidean distance matrix among samples. Then, we used this matrix in a 999-permutations PERMANOVA to assess the differences in the coloration among nests, sites, and origin, with a full nested design.

To assess the differences in the head width among sites, we performed a Linear Mixed Model (LMM), where the site was the fixed term and the nest was the random term. Tukey multiple comparisons were then performed between sites in pairs. Finally, to test the dependency of the shape on the size, we subdivided the head width data into three categories according to the 33th, 66th, and 100th percentiles, and we tested the differences among these groups by performing a 999-permutations PERMANOVA test using the same Euclidean distance matrix. Analyses were performed using the “mixOmics” [50], “vegan” [51], and “multcomp” [52] packages implemented for the 4.1.0 version of the R software [53].

## 3. Results

**Figure 3:**
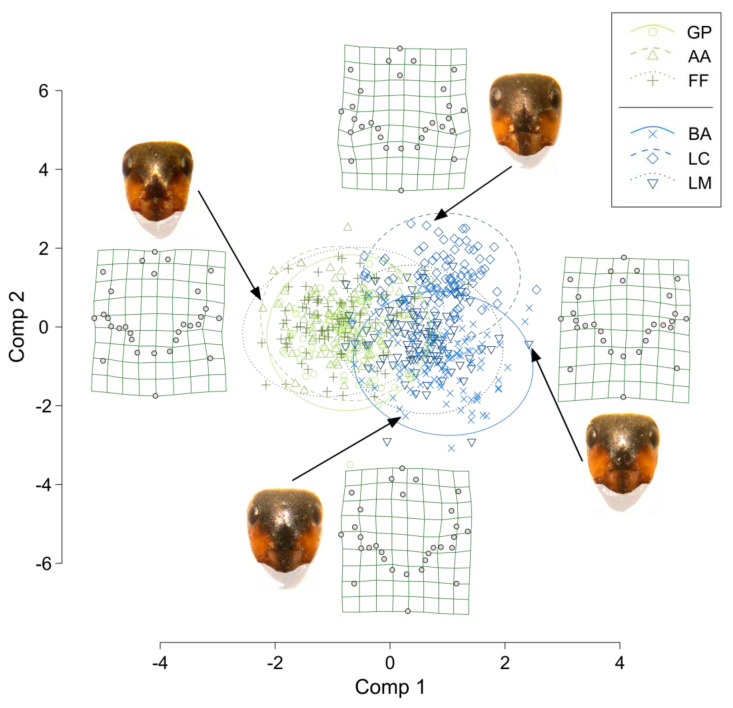
PLSDA global projection of relative warps from the facial coloration landmarks. Green grids are examples of thin-plate spline representations of four samples selected at the border of the PLSDA projection, so that they show the differences in the shape of facial coloration according to the two components. Samples are indicated by the arrows, and the picture of their face is reported aside their thin-plate spline representation. The same colored and shaped symbols are samples from the same population, and ellipses represent the 95% C.I. of each population. In the thin-plate spline grid representations, points are both landmarks and semi-landmarks (see Figure 2 for details). GP = Giovetto di Paline, AA = Avorniolo Alto; FF = Fosso Fresciaio; BA = Baradello; LC = Le Cullacce; LM = La Lama. GP and BA are the Alpine native sites.

Figure 3 shows the global PLSDA projection—with 95% CI ellipses grouping samples from different sites—and examples of thin-plate spline interpolations of four samples, close to their position in the projection. Overall, the samples from the two groups of sites having different origins were partly separated, particularly along the first component. The samples from the alpine site GP and the two derived populations AA and FF fully overlapped, whereas, on the contrary, the samples from the alpine site BA were partly separated along the second component from those of the derived populations LC and LM, but more so with LC, and LM largely overlapped with BA. The thin-plate spline representations show that along the first component, one of the most evident differences in shape was due to the length of a dark stripe below the eyes, whereas the color border around the clypeus appeared to be more angular for the samples of the left side of the graph and more rounded for samples on the right. Based on the VIP scores, we selected 14 out of the 56 relative warps, to be used in the PERMANOVA tests. These showed a significant difference between origins (df = 1, pseudoF = 34.43, *p* = 0.001), sites within an origin (df = 4, pseudoF = 8.96, *p* = 0.001), and nests within sites (df = 6, pseudoF = 4.10, *p* = 0.001).

**Figure 4:**
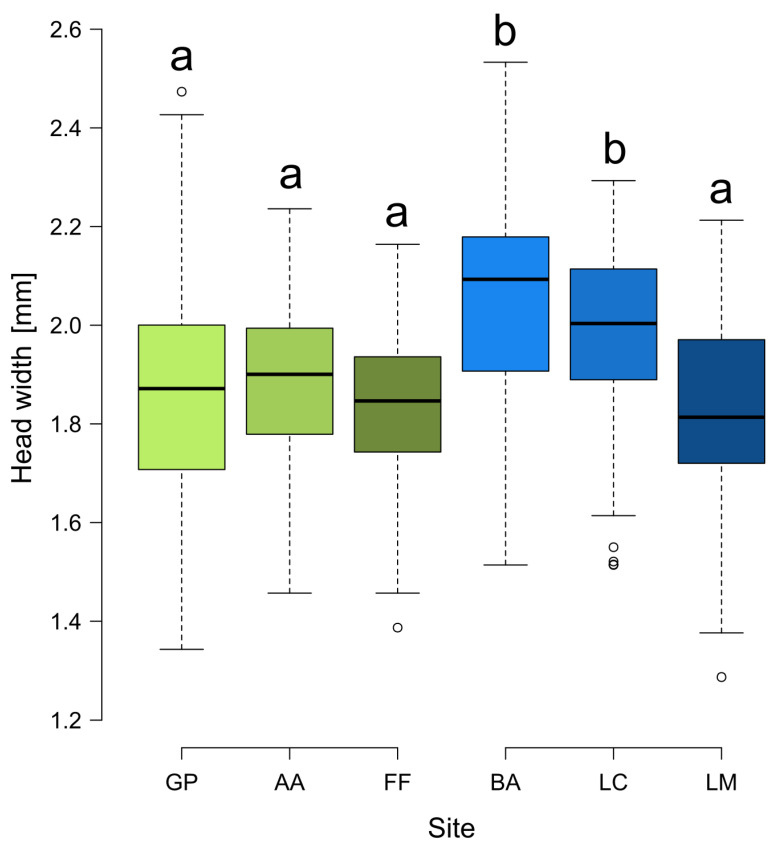
Boxplots of the head width of *Formica paralugubris* in the six sites. Letters above boxes summarize the results of Tukey’s multiple comparisons (boxes with the same letter indicate they are not significantly different from one another). Green and blue boxes represent populations derived from the Alpine sites of GP and BA, respectively. Shades of color represent the different populations and are the same of ellipses and symbols in the PLSDA projection (Figure 3). GP = Giovetto di Paline, AA = Avorniolo Alto; FF = Fosso Fresciaio; BA = Baradello; LC = Le Cullacce; LM = La Lama.

The head width was significantly different among sites (F_5,54_ = 8.012, *p* < 0.001), being higher at BA and LC than in the other sites, which were all similar (Figure 4). The difference in the shape of facial landmarks according to the size category was significant (df = 2, pseudoF = 11.94, *p* = 0.001). In the Supplementary Materials Figure S1, PLSDA projections by grouping samples per nest for each site are reported.

## 4. Discussion

The main result of our study is that nests, sites, origin, and worker size affect the shape of the facial coloration of *F. paralugubris*. The facial coloration of the ants from the alpine site GP and the two derived populations, AA and FF, appear to be similar. On the contrary, the patterns of the ants from the other alpine site, BA, and its two derived populations, LC and LM, clearly differ from that of GP, but a clear similarity occurs only between BA and LM.

The similarity between GP samples and those from the two derived populations seems to support the hypothesis of a common genetic background of the coloration pattern, with local variations between the single nests. Environmental factors, instead, seem not to have any generalized effect on coloration. The three sites are characterized by different dominant arboreal species (spruce in GP, fir in AA, and beech in FF) and climate, since in the alpine sites, the temperature is on average lower, and precipitation is higher [54]. These differences are further exacerbated by the fact that the Alpine populations inhabit forests at higher elevations (approximately from 1500 to 1700 a.s.l.) compared to the Apennine populations (approximately from 1000 to 1300 a.s.l.). The absence of well-defined differences between the three sites is quite surprising, in light of recent findings showing that the color pattern of RWAs can be a good indicator of habitat quality [30,32], and it has already been used as such in previous studies on *F. aquilonia* [55,56]. The similarity between GP and its derived populations could be explained by the reproductive biology of the studied species. In polygynous RWAs, reproduction and dispersion strategies are based on an intranidal mating system and budding—new nests established close to the main colony by a small group of queens and workers [57,58]. This leads to a slow and short-distance expansion of the population, which prevents imported populations from colonizing larger areas, keeping them isolated. It is therefore possible that the absence of genetic flux has helped to conserve the original phenotype.

On the other hand, in BA and the corresponding imported populations of LC and LM, the picture is less clear. The facial coloration of the ants is similar only between the original site and LM, although with a few differences. In LC, the differences with the other two sites are evident, suggesting that, in this case, the environmental component overcame the genetic inheritance. Since environmental conditions are very similar between LC and LM (similar forest stand, climate, and altitude), this result is tricky to interpret. One possible source of uncertainty is that we cannot be sure that the nest collection was actually performed from a single population in the Baradello mountain, since the authors did not give a detailed position except for the toponym of the sites [35]. Giovetto di Paline is included in a protected area, and a detailed record of the location of management interventions is available. Similar information is lacking for the Baradello mountain, because it has never been included in a protected area, and thus we cannot be sure that the LC population actually descends from the very same BA population we sampled, which is instead compatible with the LM population. Moreover, in the last decades, the Baradello mountain has been transformed into a ski area, with several linear clear-cuttings performed to make room for ski slopes. Such a severe habitat modification may have had a significant impact on arthropod communities due to the fragmentation of the forest habitat [59,60], with a cascade effect on the genetic structure of polydomous RWA populations [61]. Another source of uncertainty is that the Apennine populations probably underwent a bottleneck effect a few years after their introduction. According to Groppali and Crudele [62] (in Italian), most of the nest mounds introduced in the Apennine sites (20 at AA, 19 at FF, 27 and 20 at LM and LC, respectively) disappeared a few years later, and in nearly all areas, the populations were reduced to 3-6 mounds. It is therefore possible that some color patterns disappeared, causing the divergence we observe today. If available, some useful insights could come from the analysis of museum samples collected at the time of the nests’ collection before the introduction in the Apennines [34], which could unveil the coloration patterns existing in the populations at that time.

The fact that the coloration is different among nests within the same population may depend both on genetic and external factors. In the first case, nests—or subsets of them—should belong to different colonies that are genetically isolated from one another. Although *F. paralugubris* has been shown to be unicolonial at some sites in the Swiss Alps [63,64], this does not seem to be the case at the studied Apennine sites, where non-negligible intra-population aggressiveness has been recently documented [40]. On the other hand, if—as in *F. rufa*—kinship is not correlated with the color pattern variability [33], the factors influencing coloration should be prevalently external and localized, such as sun exposure, humidity, or even the occurrence of an environmental stressor. A very recent study analyzing the genetic variability of the same ants used in this study based on AFLP markers shows that the major part of the variability among populations can be explained at the individual level, and that the imported populations have an overall genetic diversity higher than the native ones [65]. This result tends to confirm the conservative heritability of the trait, which remains similar between the source and introduced populations despite the large variability recorded among nests and workers. Looking at this picture, it seems that facial coloration in this species is a polyphenic trait, whose epigenetic control is driven by local external factors, as recently hypothesized for *F. rufa* [33]. An accurate assessment of the effect of population-level localized factors and a genetic analysis assessing the relationships occurring among individuals, nests, and populations might help to shed light on this point.

We also found that the size of the ants affects the shape of their facial coloration. The variability in the coloration with the size is in line with previous studies on RWAs [30,31]. Since ant size differed among populations, in the case of allometry, the variability might be partially explained by this, despite the use of the Procrustes superimposition [66]. However, a recent study on *F. lugubris*, a sister species that is morphologically very similar to *F. paralugubris* [24], shows that the variation in the head shape with regard to the body size is isometric [67]. One of the traits that mostly appears to change among individuals is the different length of the dark stripe under the eyes. For example, long dark stripes are present in many ants from LC, which are on average also larger than in other populations, except for BA. In principle, longer black stripes mean a higher proportion of black in the face coloration. This would suggest that the larger the ants, the darker the coloration, a result in contrast with previous studies, in which small ants were darker than large ones [30]. However, we did not analyze the color of the thorax, and we cannot be sure that other parts of the body were darker or lighter. Our novel approach to the analysis of facial coloration might result in different outcomes with respect to previous studies, since it analyzes more the shape of this trait in-depth instead of its coverage percentage. Further studies using this method should be useful in exploring the potentialities of its use in intraspecific comparative analyses of similar phenotypic traits.

## 5. Conclusions

We used a landmark-based morphological approach for the first time to assess the difference in the facial coloration among introduced populations of the RWA *F. paralugubris* and their populations of origin. Except for the case of the introduced population of LM discussed above, our results suggest that this trait can be heritable, given the clear similarity between Alpine-native and introduced sites. The interesting point here is that this phenotypic trait appears to be rather conservative, since it did not change in the ants of imported populations during the six decades that elapsed from the introductions, despite the different habitats they occupy. This suggests a genetic background in expressing this trait, which needs long-lasting adaptive pressures to be modified at the population level. On the other hand, the variability shown at the nest level might be a response to local external factors, which would suggest polyphenism for this trait. Future studies might investigate how this trait is affected by very local factors around the nests, such as insolation, humidity, soil pH, or the presence of pathogens and parasites. Moreover, a detailed molecular analysis should be performed between original and introduced populations to assess how this trait is linked to the kinship and phylogeny of this species. A fascinating development of the study might concern whether and how facial coloration is implied in the social communication in these ants, for example, as a badge of health status or in recognizing conspecifics, as demonstrated for several wasp species [68,69,70]. Our result showing a nest-level difference in this phenotypic trait is a hint that appears to go in this direction.

## Data Availability

Data available on request.

## Supplementary Materials

The following supporting information can be downloaded at: https://www.mdpi.com/article/10.3390/insects13121137/s1, Figure S1: PLSDA projections of relative warps from facial coloration shape of ant workers in each population. Nests are represented by different colors and symbols. Ellipses are 95% C.I. GP = Giovetto di Paline, AA = Avorniolo Alto; FF = Fosso Fresciaio; BA = Baradello; LC = Le Cullacce; LM = La Lama. GP and BA are the Alpine native populations.
