# Supplementary material for: Comparative Analysis of Facial Coloration between Introduced and Source Populations of the Red Wood Ant Formica paralugubris
Source: Insects. 2022 Dec 9;13(12):1137. doi: 10.3390/insects13121137 (PMC9787359; doi:10.3390/insects13121137)

Figure S1. PLSDA projections of relative warps from facial coloration shape of ant workers in each population. Nests are represented by different colors and symbols. Ellipses are 95% C.I. GP = Giovetto di Paline, AA = Avorniolo Alto; FF = Fosso Fresciaio; BA = Baradello; LC = Le Cullacce; LM = La Lama. GP and BA are the Alpine native populations.

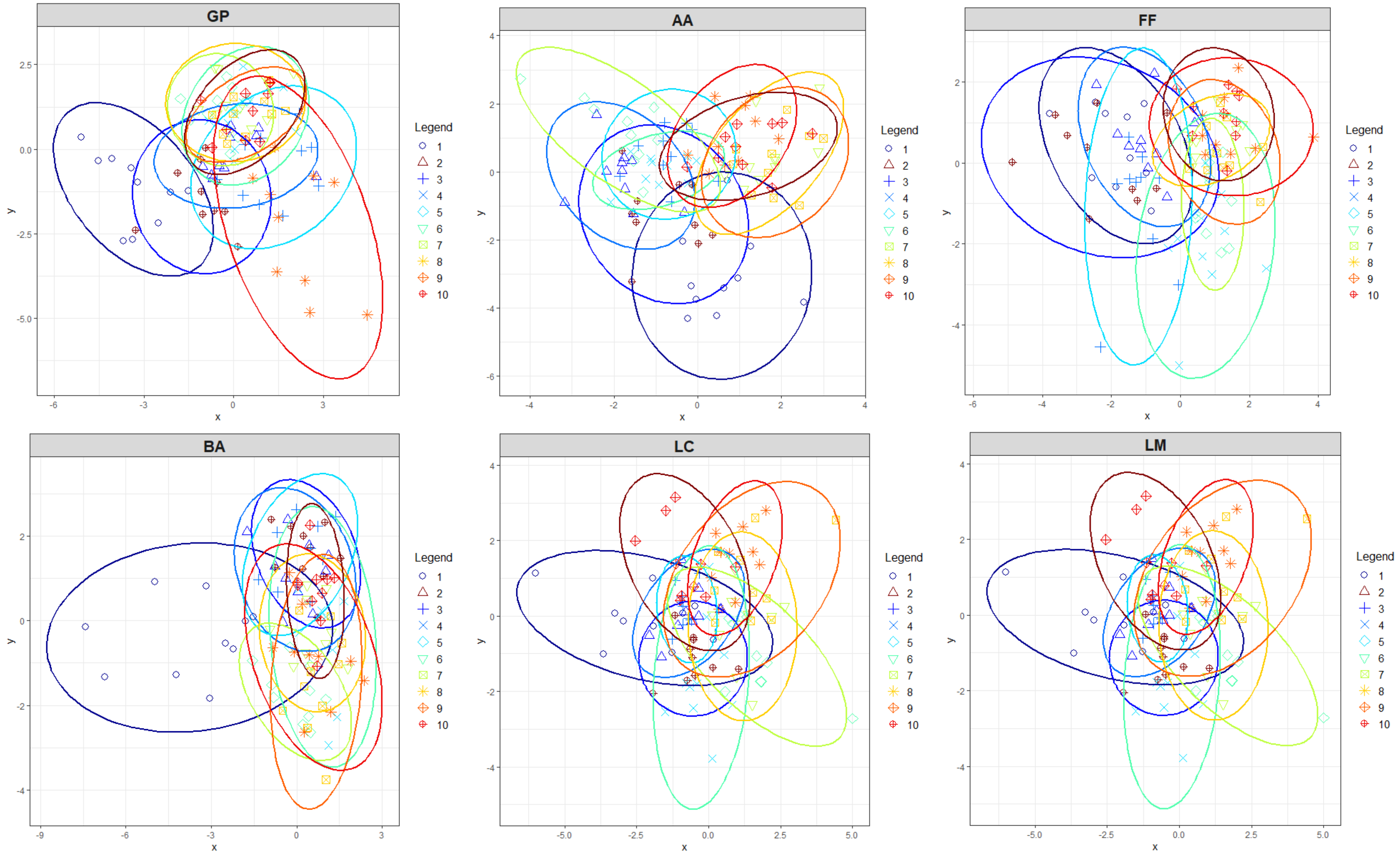

Supplement: Supplementary file 1 [file insects-13-01137-s001.zip › insects-2069218-supplementary.pdf]
